# Malaria outbreak investigation and contracting factors in Simada District, Northwest Ethiopia: a case–control study

**DOI:** 10.1186/s13104-019-4315-z

**Published:** 2019-05-17

**Authors:** Baymot Workineh, Fantahun Ayenew Mekonnen, Mekonnen Sisay, Kedir Abdela Gonete

**Affiliations:** 1 Department of Epidemiology and Biostatistics, Institute of Public Health, College of Medicine and Health Sciences, University of Gondar, Gondar, Ethiopia; 2 Department of Human Nutrition, Institute of Public Health, College of Medicine and Health Sciences, University of Gondar, Gondar, Ethiopia

**Keywords:** Malaria, Outbreak, Investigation, Contracting factors, Case control, Simada, District, Ethiopia

## Abstract

**Objective:**

The aim of this study was to assess the occurrence of malaria outbreak and investigate contracting factors of malaria in Simada District, Northwest Ethiopia. A single observation original research.

**Results:**

Among the total 54 cases, 44 (81.5%) of them were confirmed malaria cases. The average attack rate was 20 per 100 and slide positivity rate was 81.5%. People in the age group of 5–14 years were most affected with an attack rate of 37%. Presence of water bodies for mosquito breeding inside less than 1 km radius (AOR = 3.32, 95% CI 1.18–9.34), no knowledge on transmission, prevention and control mechanisms of malaria (AOR = 4.36, 95% CI 1.64, 12.23), not using Insecticide Treated Bed Net (AOR = 5.85, 95% CI 1.94, 17.54) and absence of environmental control (AOR = 10.01, 95% CI 2.94, 33.33) were factors associated with malaria outbreak.

**Electronic supplementary material:**

The online version of this article (10.1186/s13104-019-4315-z) contains supplementary material, which is available to authorized users.

## Introduction

Malaria is endemic throughout the tropical areas of the world with the highest prevalence found in sub-Saharan Africa, India, and Southeast Asia [1]. There were about 219 million estimated malaria cases globally in 2017. About 200 million or 92% of malaria cases in 2017 were in the WHO African Region, followed by the WHO South-East Asia Region with 5% of the cases [2]. In 2017, there were an estimated 435 000 deaths from malaria globally. The WHO African Region accounted for 93% of all malaria deaths in 2017 [2].

In Ethiopia, about 68% of the total population resides in areas with high malaria risk [3] and 2,174,707 cases and 662 deaths due to malaria were reported in 2014–2015 with case fatality rate (CFR) of 0.03% [4]. There was, 1,127,241 malaria cases, out of a total population of 19,867,817 in Amhara Region in 2012 [5]. Low land areas (below 2000 meters of altitude) in Ethiopia are a place where more people are affected by malaria than high landers (above 2000 meters of altitude) [6]. The major malaria vector in Ethiopia is Anopheles arabiensis and the most dominant malaria parasites are *Plasmodium falciparum* (PF) and *Plasmodium vivax* (PV) [7]. Early diagnosis and prompt treatment, selective vector control, use of insecticide-treated mosquito nets (ITNs), and environmental management are the four main intervention strategies that are being applied in Amhara Region and Simada District, Workaye Kebele to combat malaria [8].

According to the Ethiopian national malaria indicator survey result 55.2% of households have at least one mosquito net and 38.2% of under five children had utilized ITNs [9]. In Amhara Region, 34.7% and 16.6% of households owned at least one net and one LLIN respectively. The mean numbers of nets and LLIN per house in Amhara Region were 0.5 and 0.3 respectively. Among those who had LLIN, only 12.5% were slept under LLIN or utilized it properly. From these who properly used LLIN, about 14.5% and 14.6% were under five children and pregnant women aged 15 to 49 years old respectively [10].

Population residing in malaria endemic areas of Amhara Region are affected during planting and harvesting seasons, cutting down productive capacity. It is also associated with loss of earnings, low school attendance, and high treatment costs and patient overload on health facilities [4, 11].

Therefore, this study aimed at investigating the causes of the outbreak in Simada District and identifying factors associated with contracting malaria. Besides, it also tried to describe outbreak trends by person place and time and thus providing feasible recommendations of the finding to control and preventive measures towards malaria outbreak.

## Main text

### Methods

**Fig. 1:**
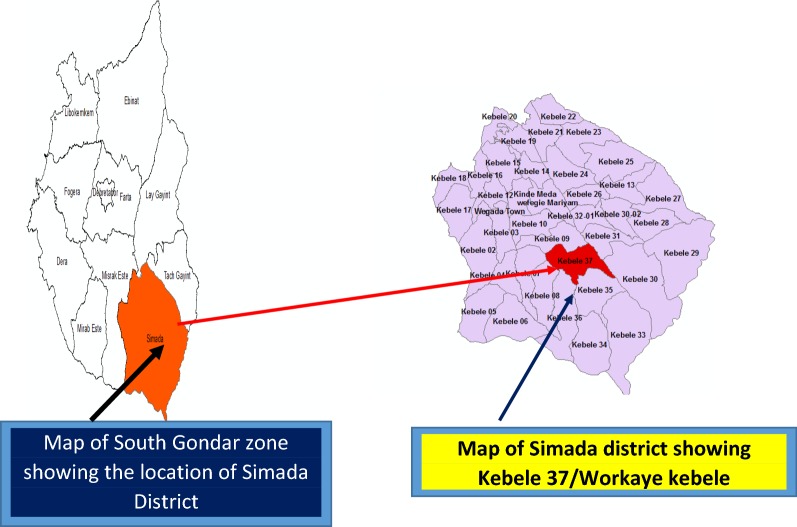
Map of investigation area, Workaye Kebele, Simada District, Amhara Region, Ethiopia 2016 (source: Workaye Kebele administration office)

The study was conducted in Workaye Kebele (the smallest administration unit in Ethiopia) from Jun 09 to 20/2016. Workaye is found in Simada District, Amhara region, Ethiopia. It is located 650 km far from Addis Ababa, the capital city of Ethiopia. The total population residing in the Kebele was estimated to be 7725. Of which 51% were males and 49% were females. The study setting is located in dissected landscapes of Abay-Beshilo Basin of Simada District where land degradations, drought, food insecurity and famine are serious problems mainly since 1980s. It is totally included in the Abay River Basin. The elevations of the study area ranges from 854 m to 1500 m above sea-level. The temperature ranges from 24  to  28 °C and rainfall from 200–900 mm (Fig. 1) [12].

On June 2016, a rumor of malaria outbreak in Workaye Kebele was notified to South Gondar Zone health department by Simada District health office. To verify the occurrence of the outbreak current data was compared with threshold. The 2016 data was compared with the average weekly number of cases during 2011–2015 to determine whether the epidemic threshold had been crossed.

Un-matched case–control study was used to identify risk factors of malaria outbreak from June 09 to 20, 2016. Data was collected by checklist on malaria outbreak risk factors in four Villages of Workaye Kebele. Selected cases and controls were interviewed and observation also was made on man mad water -holding bodies close to home and presence of anopheles’ larvae in stagnant water.

Those people with and without malaria signs and symptoms for less than 2 weeks were selected from the community as cases and controls in 1:2 ratio. They were selected regardless of their age, gender, educational status, physiological status and socio-economic status. Epi Info version 3.5.1 and SPSS version 20 were used to describe the disease and analyze associated risk factors. Bi-variable and multivariable analysis and Odds Ratio (OR) with its corresponding 95% Confidence Interval (CI) were calculated to determine the significance of risk factors with the outbreak.

The sample size was calculated using EPI-Info statistical package. The total calculated sample size was 162 (54 cases and 108 controls).

#### Ethical considerations

Ethical approval was obtained from Ethical review board of University of Gondar. Permission letter was also secured from Simada District health office. In addition, written consent was obtained from study participants after providing a clear information about the overall objectives of the study.

### Results

#### Socio demographic characteristics

The study included 71 (43.8%) males and 91 (56.2%) females. Most of the participants were greater than 15 years. Concerning their ethnicity and religion all 162 (100%) of them were Amhara and Orthodox Christian. Seventy-two participants (44.4%) were married and 61 (37.7%) were single (Table 1).

**Table 1:** Socio demographic characteristics of the study participants in Workaye Kebele, Simada District, Northwest Ethiopia, 2016

Variable	Cases		Controls	
	N	%	N	%
Sex				
M	28	51.9	43	39.8
F	26	48.1	65	60.2
Marital status				
Single	32	59.3	29	26.9
Married	15	27.8	57	52.8
Widowed/divorced	7	13	22	20.3
Educational status				
N/A	8	14.8	8	7.4
Unable to read and write	11	20.4	36	33.3
Able to read and write	8	14.8	20	18.5
Primary	27	50	38	35.2
Secondary and above	0	0	6	5.6
Ethnicity				
Amhara	54	100	108	100
Others	0	0	0	0
Religion				
Orthodox	54	100	108	100
Others	0	0	0	0
Occupation				
N/A	10	18.5	9	8.3
Farmer	16	29.6	63	58.3
Student	28	51.9	36	33.3

#### Descriptive epidemiology by person

A total of 227 malaria cases were examined by RDT/Microscopy. Of which 154 (67.8%) were positive for malaria. Age group 5–14 years and females were the most affected groups with an attack rate of 37 and 29 per 100 population. The median age of cases and controls was 18 and 20 years. Average attack rate was 28 per 100 population with no malaria death. The positivity rate among randomly tested 50 fever cases was found to be 92% confirmed by slide microscopy which confirmed the existence of an outbreak according to the national guideline threshold of > 50% (Additional file 1: Table S1).

Due to the increased number of malaria cases from Workaye Kebele the threshold of Yekosa health center was crossed at WHO- Epi- Week 22 (Additional file 2: Figure S1).

Due to increased number of malaria cases from Welekoch, Tig mender, Edari mender and Addis Amba Villages, the threshold of Workaye health post was crossed at WHO- Epi- Week 22 (Additional file 3: Figure S2).

#### Descriptive epidemiology by place

Among the total population who were at risk of malaria, 280/835 (33.5%), 215/835 (25.8%), and 155/835 (18.6%) were from Addis Amba, Welekoch and Tig mender villages respectively. *PF* followed by *PV* were the most dominant species responsible for the outbreak. Positivity rate was higher in Addis Amba village (28.2%) as compared to the rest. The attack rate was more in Tig mender and Welekoch Villages, 27 and 21 per 100 population (Additional file 4: Table S2).

##### Laboratory

Microscopy and RDT laboratory tests were done for 227 malaria cases in Yekosa health center and Welekoch health posts. Of the total tested cases, 154 (67.8%) were positive for malaria. Among the positive cases, 104 (67.5%) were *PV* and 50 (32.5%) *PF* (Additional file 5: Figure S3).

#### Analytical investigation

In the Bi-vairiate logistic regression, factors that were significantly associated with increased risk of malaria case were traveling history with risk effect of significantly (OR = 6.53, CI 2.19–19.5), presence of artificial water holding containers near to the home (OR = 6.8, CI 3.3–14), presence of intermittent rivers with 1 km radius (OR = 10, CI 4.4–22.7) and staying outside the home overnight (OR = 4.6, CI 2.16–9.78); whereas knowledge on transmission, prevention and control of malaria (OR = 0.19, CI 0.1–0.47), utilization of LLINs (OR = 0.2, CI 0.096–0.417) and environmental control (OR = 0.22, CI 0.1–0.473) were protective factors. While, presence of artificial water holding bodies near to the home, presence of intermittent rivers within 1 km radius, knowledge on malaria transmission, prevention and control, LLINs utilization, and environmental control were factors remained associated with malaria contracting during multivariate analysis.

Accordingly, people who were living in households where artificial water holding bodies were 3.3 times more at risk of contracting malaria than their counterparts (AOR = 3.32, 95% CI 1.18, 9.30). Likewise, presence of intermittent rivers closes to the community within 1 km distance increased the likelihood of getting malaria than those far away from it (AOR = 4.72, 95% CI 1.6, 13.65).

Similarly, people who did not used ITNs were at higher risk of developing malaria than who used (AOR = 5.85, 95% CI 1.94, 17.54). Furthermore, those who did not controlled their living environment were 10 times at higher risk of contracting the disease (AOR = 10.01, 95% CI 2.94, 33.33) and higher odds of malaria were noted among those who had no knowledge on malaria transmission, prevention and control mechanisms (AOR = 4.36, 95% CI 1.64, 12.23) (Table 2).

**Table 2:** Multivariate analysis of risk factors for malaria outbreak Workaye Kebele, Simada District, Northwest Ethiopia, 2016

Variables	Cases N = 50 (%)	Controls N = 100 (%)	COR	95% CI		AOR	95% CI		*P* value
				Lower	Upper		Lower	Upper	
Traveling history									
Yes	13	5	6.53	2.19	19.5	2.21 1	0.84	3.86	0.12
No	41	103	1						
Presence of artificial water holding bodies near to the home									
Yes	35	23	10	4.4	22.7	3.32 1	1.18	9.3	< 0.001
No	19	85	1						
Presence of intermittent rivers with 1 km radius									
Yes	45	36	5.41	2.11	10.00	4.72 1	1.6	13.65	0.004
No	9	72	1						
Knowledge on transmission, prevention and control of malaria									
Yes	17	81	1			1 4.36	1.64	12.23	0.005
No	37	27	5.00	2.40	10.42				
Staying outside the home overnight									
Yes	24	16	6.8	3.3	14	3.23 1	0.78	4.32	0.07
No	30	92	1						
Utilization of LLINs									
Yes	27	90	1			1 5.85	1.94	17.54	0.002
No	27	18	4.61	2.16	9.78				
Environmental control									
Yes	11	58	1			1 10.01	2.94	33.33	< 0.001
No	43	50	4.54	2.11	10.00				

#### Environmental assessment

Mesk River and Kugena dam were found in Workaye Kebele nearest to affected Villages. As it was observed, there were multiple cracking sites favorable for mosquito breeding and mosquito larvae was seen on the river. Forty-two households from affected Villages were randomly selected and visited. According to the visit, water contained well, stagnant water and improper use of drainage system were observed. All households had at least one LLINs in their house. More than half (53.7%) of them used LLINs and 32.5% used it properly. Among those use did not used LLINs, most of them used it for other purposes, dirty, not properly hung at sleeping space and put under box. There were larvae of mosquitoes in observed stagnant water. According to clients’ response, indoor residual spray was not performed for the last 1 year in all outbreak affected households.

#### Interventions done

- Focal spray was performed for all affected Villages.
- Dam on the river was opened.
- Education was given on prevention and control measures of malaria.
- Health professionals were assigned to affected Villages for early case management at community and health facility level.
- Mass fever treatment was given.
- Contributing factors for malaria outbreak were rapidly assessed in the environment and control of breeding sites was done.
- Surveillance system was strengthened.

### Discussion

Based on 5 years’ epidemiological records of malaria cases, the study findings confirmed existence of malaria epidemic in the study area. Malaria outbreak began in the area was in line with other areas of Ethiopia and the national data in terms of time period when increased malaria cases reported. April to December is a period in which malaria epidemic is commonly happened in different regions of Ethiopia [13].

The number of malaria cases reported were doubled compared to the prior year in WHO epidemic week 22/2016. The peak magnitude of malaria cases showed in week 26/2016. Index case from Epi-curve and threshold graph showed outbreak response was started late.

Age group of 5–14 years and females were more affected with AR of 37 and 29 per 100 population. Studies in India [14], Ethiopia [15] and Zimbabwe [16] showed children and females were more attacked by malaria. The reason may be children have lower immunity and adult women do more activities that exposed them to mosquito breeding sites.

Households found within 1 km radius had odds of 4.72 of contracting malaria as compared to those far away. This is due to presence of multiple cracked sites of Mesk River and was favorable for mosquito breeding. A study in Zimbabwe reported the association of staying close to such water sources and contracting malaria [16]. Hence, there should be sustainable integrated and coordinated malaria prevention and control measures and proper water management to eliminate mosquito breeding sites [17].

Stagnant water formed following heavy rainfall creates a conducive breeding site for mosquitos and is a cause for malaria epidemics [18]. Similarly, our study recognized that people found close to the stagnant water were 3.3 times more affected than those who lived far from it. This finding is supported by a report from Afar, Ethiopia [19].

A study in Kenya showed household ownership of a mosquito net was associated with lower risk of malaria impact and transmission [20]. Studies from Zimbabwe and Beitbridge reported that not having an ITN hanged in the room was significantly associated with contracting Malaria [16, 21]. Findings of this study also revealed similar results.

Awareness about malaria is an important aspect to prevent disease exposure and its related negative health consequences [22, 23]. A study in Ethiopia revealed that general knowledge about malaria helped the community to prevent and control the disease thereby, it has a significant contribution in the reduction of burden of malaria [24]. Similarly, in the current study, having no awareness on means of transmission and prevention and control measures of malaria was a risk of contracting the disease.

The likelihood of occurrence of the outbreak in the current study area increased tenfolds in poorly controlled environment. Similar study finding was also reported from other part of Ethiopia [15].

### Conclusion

Malaria outbreak was confirmed in Workaye Kebele. Age group of 5–14 years and females were more affected by malaria outbreak. Lack of awareness on malaria transmission and control, presence of rivers, dams, stagnant water, poor bed net utilization, presence of artificial water bodies and poor environmental control were significantly associated with the occurrence of malaria outbreak.

Therefore, community awareness regarding ITN utilization and malaria prevention and control mechanism should be created. Beside, utilization of bed net should be monitored and optimized. Regular indoor residual spray should have to be done before rainy season twice per year. Removal of potential mosquito breeding sites should be conducted regularly.

### Limitations of the study

Knowledge of study population regarding importance, duration, and care of insecticide treated bed nets and availability of chemical spray in the District health office stock and the reason why indoor chemical spray were not assessed.

## Additional files

**Additional file 1: Table S1.** Malaria Attack Rate per 100 population by age and sex in Workaye Kebele, Simada District, Northwest Ethiopia. This data shows which gender and age group is more affected among the total population in Wokaye Kebele (malaria attack rate per 100 population with respect to age and sex).

**Additional file 2: Figure S1.** Threshold of Malaria in Yekosa health center in Workaye Kebele, Simada District, Northwest Ethiopia. It shows number of malaria cases in Yekosa health center in comparison to WHO week and 2015 (WHO week that showed malaria threshold comparing, cases of 2015 with 2016).

**Additional file 3: Figure S2.** Malaria cases threshold in Workaye health post, Workaye Kebele, Simada District, Northwest Ethiopia. It shows number of malaria cases in Workaye health post in comparison to WHO week and 2015 (WHO week that showed malaria threshold comparing, cases of 2015 with 2016).

**Additional file 4: Table S2.** Malaria attack rate by Village in Workaye Kebele, Simada District, Northwest Ethiopia. This data is about the distribution of malaria across affected Villages in Workaye Kebele (distribution of total malaria cases in four Villages and attack rate per 100 population among the total population).

**Additional file 5: Figure S3.** Epidemic curve of malaria outbreak in Workaye Kebele, Simada District, Northwest Ethiopia. This data provides an information about the increment of malaria cases as compared to the previous number of cases, the time when the index case was identified, date of onset, notification time to Zonal public health emergency management and the time when the investigation was started.

## Data Availability

Data are available from the corresponding author on reasonable requests.

## Abbreviations

AOR: adjusted odd ratio
AR: attack rate
CFR: case fatality rate
CI: confidence interval
COR: crude odd ratio
IRS: indoor residual spray
ITN: insecticide-treated Mosquito Net
KM: kilo meter
LLIN: long-lasting insecticide-treated nets
OR: odd ratio
PF: *Plasmodium falciparum*
PHEM: Public Health Emergency Management
PV: *Plasmodium vivax*
RDT: Rapid Diagnostic Test
RRT: Rapid Response Team
SPSS: Statistical Package for Social Sciences
WHO: World Health Organization
