# Supplementary material for: Malaria outbreak investigation and contracting factors in Simada District, Northwest Ethiopia: a case–control study
Source: BMC Res Notes. 2019 May 17;12:280. doi: 10.1186/s13104-019-4315-z (PMC6525450; doi:10.1186/s13104-019-4315-z)
Supplement: Supplementary file 1 — Additional file 1: Table S1. Malaria Attack Rate per 100 population by age and sex in Workaye Kebele, Simada District, Northwest Ethiopia. This data shows which gender and age group is more affected among the total population in Wokaye Kebele (malaria attack rate per 100 population with respect to age and sex). [file 13104_2019_4315_MOESM1_ESM.docx]

**Table S1:** **Malaria Attack Rate per 100 by age and sex**

| Variables | Total population | Number of cases | Attack rate per 100 population |
| --- | --- | --- | --- |
| Sex | | | |
| Male | 423 | 109 | 26 |
| Female | 412 | 118 | 29 |
| Age group | | | |
| <5 | 113 | 15 | 13 |
| 5-14 | 243 | 88 | 37 |
| >15 | 479 | 124 | 26 |
