# Supplementary material for: Malaria outbreak investigation and contracting factors in Simada District, Northwest Ethiopia: a case–control study
Source: BMC Res Notes. 2019 May 17;12:280. doi: 10.1186/s13104-019-4315-z (PMC6525450; doi:10.1186/s13104-019-4315-z)
Supplement: Supplementary file 3 — Additional file 3: Figure S2. Malaria cases threshold in Workaye health post, Workaye Kebele, Simada District, Northwest Ethiopia. It shows number of malaria cases in Workaye health post in comparison to WHO week and 2015 (WHO week that showed malaria threshold comparing, cases of 2015 with 2016). [file 13104_2019_4315_MOESM3_ESM.docx]

**Figure S2: Malaria cases threshold of Workaye health post**
