# Supplementary material for: Malaria outbreak investigation and contracting factors in Simada District, Northwest Ethiopia: a case–control study
Source: BMC Res Notes. 2019 May 17;12:280. doi: 10.1186/s13104-019-4315-z (PMC6525450; doi:10.1186/s13104-019-4315-z)
Supplement: Supplementary file 4 — Additional file 4: Table S2. Malaria attack rate by Village in Workaye Kebele, Simada District, Northwest Ethiopia. This data is about the distribution of malaria across affected Villages in Workaye Kebele (distribution of total malaria cases in four Villages and attack rate per 100 population among the total population). [file 13104_2019_4315_MOESM4_ESM.docx]

**Table S2: Malaria attack rate by Village**

| Name of Village | Total populations | Total Cases | Attack rate/100 populations |
| --- | --- | --- | --- |
| Welekoch | 215 | 63 | 21 |
| Tig mender | 155 | 57 | 27 |
| Addis Amba | 280 | 64 | 14 |
| Edari mender | 185 | 43 | 16 |
