# Supplementary material for: Malaria outbreak investigation and contracting factors in Simada District, Northwest Ethiopia: a case–control study
Source: BMC Res Notes. 2019 May 17;12:280. doi: 10.1186/s13104-019-4315-z (PMC6525450; doi:10.1186/s13104-019-4315-z)
Supplement: Supplementary file 5 — Additional file 5: Figure S3. Epidemic curve of malaria outbreak in Workaye Kebele, Simada District, Northwest Ethiopia. This data provides an information about the increment of malaria cases as compared to the previous number of cases, the time when the index case was identified, date of onset, notification time to Zonal public health emergency management and the time when the investigation was started. [file 13104_2019_4315_MOESM5_ESM.docx]

**Figure S3: Epidemic curve of malaria outbreak**
